# MetNet Online: a novel integrated resource for plant systems biology

**DOI:** 10.1186/1471-2105-13-267

**Published:** 2012-10-15

**Authors:** Yves Sucaet, Yi Wang, Jie Li, Eve Syrkin Wurtele

**Affiliations:** 1 Dept of Genetics, Development and Cell Biology, Iowa State University, Ames, IA, USA; 2 Interdepartmental Program in Bioinformatics & Computational Biology, Iowa State University, Ames, IA, USA

## Abstract

**Background:**

Plants are important as foods, pharmaceuticals, biorenewable chemicals, fuel resources, bioremediation tools and general tools for recombinant technology. The study of plant biological pathways is advanced by easy access to integrated data sources. Today, various plant data sources are scattered throughout the web, making it increasingly complicated to build comprehensive datasets.

**Results:**

MetNet Online is a web-based portal that provides access to a regulatory and metabolic plant pathway database. The database and portal integrate Arabidopsis, soybean (*Glycine max*) and grapevine (*Vitis vinifera*) data. Pathways are enriched with known or predicted information on sub cellular location. MetNet Online enables pathways, interactions and entities to be browsed or searched by multiple categories such as sub cellular compartment, pathway ontology, and GO term. In addition to this, the “My MetNet” feature allows registered users to bookmark content and track, import and export customized lists of entities. Users can also construct custom networks using existing pathways and/or interactions as building blocks.

**Conclusion:**

The site can be reached at http://www.metnetonline.org. Extensive video tutorials on how to use the site are available through http://www.metnetonline.org/tutorial/.

## Background

Plants are an increasingly facilitating and augmenting (quality of) human life and plant systems biology resources exist in a variety of locations [1]. Those researchers interested in a particular biological mechanism should be able to easily find and access all the data they need. Eliminating the need to go through the difficult process of shifting data from various databases from different platforms provides a strong incentive to create better, more integrated and easily accessible integrated plant portals.

A biological network database needs to capture and represent biological relationships in many ways. MetNet consists of a suite of software tools that specialize in different areas of systems biology [2-11]. Our database currently contains information about three plant species (Arabidopsis, Soybean and Grapevine). In the future we plan to also incorporate *Saccharomyces cerevisiae* (for model comparison) and *Hypericum* spp. MetNetDB is the underlying database and draws the majority of its information from external databases (Table 1 – also available on our website). In addition to the retrieved information, a number of signal transduction pathways and biological entities have been manually curated with input from exper biologists.

**Table 1:** Data sources of MetNet

**Database**	**Format**	**Ref**	**Information retrieved**
AraCyc	Plain text files organized according to frame data model	[14]	Pathways, interactions and biomolecules participated in. Name, synonyms, references, comments. Majority metabolic pathways in MetNetDB come from AraCyc
AGRIS	Plain text files organized according to simple graph model	[15]	Transcription network, references and binding sites of individual transcriptional factors
GO	MySQL dump files organized according to acyclic directed graph data model	[16]	The whole copy of gene ontology database
TAIR	Plain text files (Tabular data)	[18]	Affymetrix array elements and their corresponding LocusID mapping, Unitprot ID, TargetP location of polypeptides, loci of each AraCyc pathway
MapMan	Excel files (Tabular data)	[19]	Gene annotation, MapMan BIN ID, gene function category
BioCyc open chemical compound database	Plain text files organized according to frame data model	[37]	UNQUE-ID, synonyms
ChEBI	MySQL dump organized according to directed graph data model	[38]	ChEBI ID, formula, molecular weight, IUPAC, SMILES
PubChem	XML files organized according to object data model	[39]	PubChem CID, synonyms
NCI	Structure data format according to object data model	[40]	Synonyms, CAS registry number
KEGG	Plain text files (for compounds) organized according to object data model	[41]	Synonyms
SUBA	Excel file	[42]	Protein subcellular location including experiment verified and software predicted
PPDB	Tabular data	[22]	Curated protein subcellular location, especially those in plastid
AMPDB	Tabular data	[24]	Mitochondrion proteins, the subcellular location comes from computational prediction
AtNoPDB	Tabular data	[26]	Nucleolar proteins, subcellular location comes from prediction and experiments
AraPerox	Plain text	[25]	Putative proteins in peroxisomes. Subcellular location comes from literature and computational prediction
plprot	Plain text files organized according to object data model	[23]	Subcellular location comes from TargetP prediction
BRENDA	Plain text files organized according to object data model	[43]	Enzyme’s interaction, substrate, product, activator, inhibitor, synonyms, metal ions, references
MetNet curator	Manually curation		All, with focus on signal transduction information
AtPID	Excel spreadsheet	[44]	Protein-Protein interaction data
EcoCyc	Plain text files organized according to frame data model	[45]	Pathways, interactions and biomolecules participated in. Name, synonyms, references, comments.
VitisNet	SBML files made with CellDesigner	[46]	Manually constructed pathways based on draft genome sequence

MetNet Online features are compared with those of several other databases (Table 2 and Additional file 1: Table S1). MetNet is unique in a number of ways. MetNet Online provides an easy-to-use front-end web interface and combines several important features to provide a distinctive platform. First, metabolism, signalling, and transcriptional pathways are fully integrated into a single network. Second, a sub cellular location layer (obtained via manual curation and/or information from external databases) overlays the pathways. Third, a protein-protein interaction layer extends pathway information. Fourth, the website allows for customized views of any data: (meaning any combination of pathways and interactions can be combined into a new network). Fifth, MetNet Online has a “My MetNet” component, which operates similarly to “My NCBI”. Users can keep track of (bookmark) their favourite entities as well as lists of entities of particular interest (e.g., lists of genes up-regulated in a given mutant, or metabolites derived from cytosolic acetyl-CoA). User lists can be exported to other tools. Finally, the search function is sufficiently intelligent to recognize synonyms (e.g., “water” is listed amongst the search results whether one searches for “H2O” or “water”).

**Table 2:** Comparing MetNet Online with other resources

**Feature**	**Location**	**Pathway count**	**Organisms**
MetNet	http://www.metnetonline.org	966	*Arabidopsis thaliana, Glycine max, Vitis vinifera*
KEGG [47]	http://www.genome.jp/kegg/pathway.html	422	Several phylums (>1700)
PlantCyc [48]	http://pmn.plantcyc.org/PLANT/class-tree?object=Pathways	898	Phylums Clorophyta and Streptophyta (>360)
WikiPathways [49]	http://www.wikipathways.org/	247	Some phylums (22)
Gramene [50]	http://www.gramene.org/pathway/	5960	Phylums Clorophyta / Streptophyta (>360); and *Escherichia coli*

In order to enable users to easily analyse network data and customized content in MetNet Online, we provide different ways to export data (including Graphviz .dot, SBML, and XGMML) to facilitate data-flow to external applications. For bioinformatics software developers, a separate application programming interface (API) is provided [12]. MetNet Online is complementary to other community resources and provides a starting point for researchers to develop new hypotheses about biological function [13].

## Implementation

MetNetDB is a MySQL database ( http://www.mysql.com) that serves as the central repository for all MetNet applications [7]. MetNetDB uses a labelled graph model to represent biological networks and complex internal relationships. In this data model, an entity, which represents a biomolecule with a subcellular location, is mapped to a node in the labelled graph and all of its properties are assigned to the label of that node. An interaction is also represented as a node in the model. Edges represent the relationship between an entity and an interaction. The labelled graph model provides flexibility to integrate any types of biological data from any external source. A complete list of data sources is available in Table 1.

MetNetDB provides a wide variety of data, including metabolic pathways, transcriptional regulatory networks, gene annotations, protein localization information, and metabolite annotations. Supplementary data may be added manually: curated pathway data is submitted after approval from researchers who are experts in their field, following the AraCyc model of ‘expert users’. In most cases, curated data is used to enrich the database with signal transduction interactions.

For Arabidopsis, the best annotated of the networks, most of the metabolic pathways are imported from AraCyc [14]. Regulatory information is obtained from AGRIS [15], a regulatory interaction database. AGRIS contains information on: binding site of promoters, loci of associated transcriptional factors, and corresponding references. A full copy of Gene Ontology [16,17] and TAIR gene annotations [18] are incorporated. MetNetDB integrates MapMan [19] bin annotations, which have gene annotations and functional categories of the gene products. In the future, information on regulons for Arabidopsis genes [9]will be available, too.

MetNetDB includes subcellular localization data from several sources. When other information is unknown, locations are assigned from the protein sequences based on sequence similarity (TargetP ( [20]). This method is superseded in MetNetDB by experimental information, when available, from Arabidopsis protein localization databases that collect data from a combination of experimentation, literature, and predictions. Specialized resources are used for particular organelles: PPDB [21,22], plprot [23], AMPDB [24], AraPerox [25], and AtNoPDB [26]. including . PPDB (Friso et al., 2004) and plprot (Kleffmann et al., 2006) contain plastid proteins of Arabidopsis and other plants. AMPDB (Heazlewood and Millar, 2005) contains Arabidopsis mitochondrial protein data. AraPerox (Reumann et al., 2004) contains peroxisomal Arabidopsis proteins. AtNoPDB (Brown et al., 2005) contains Arabidopsis nucleolar proteins.

MetNetDB collects literature references from several sources. AraCyc and AGRIS provide references for interactions. ChEBI provides references for chemical compounds. TAIR provides references for genes and corresponding RNAs and proteins. Others references are input by curators. Our internal curator tool integrates with PathBinder [5,7] to allow searching PubMed and adding selected biological references.

Soybean data was inferred from microarray and sequence annotation with Arabidopsis as a starting point for further curation. Based on Arabidopsis network data, Locus IDs were converted to corresponding Soybean (UniGene) IDs.

Grapevine pathways were all manually constructed by field experts using CellDesigner [27]. A special plugin [28] was subsequently used to transfer the data to MetNetDB.

## Results

### The MetNet Online portal

**Figure 1:**
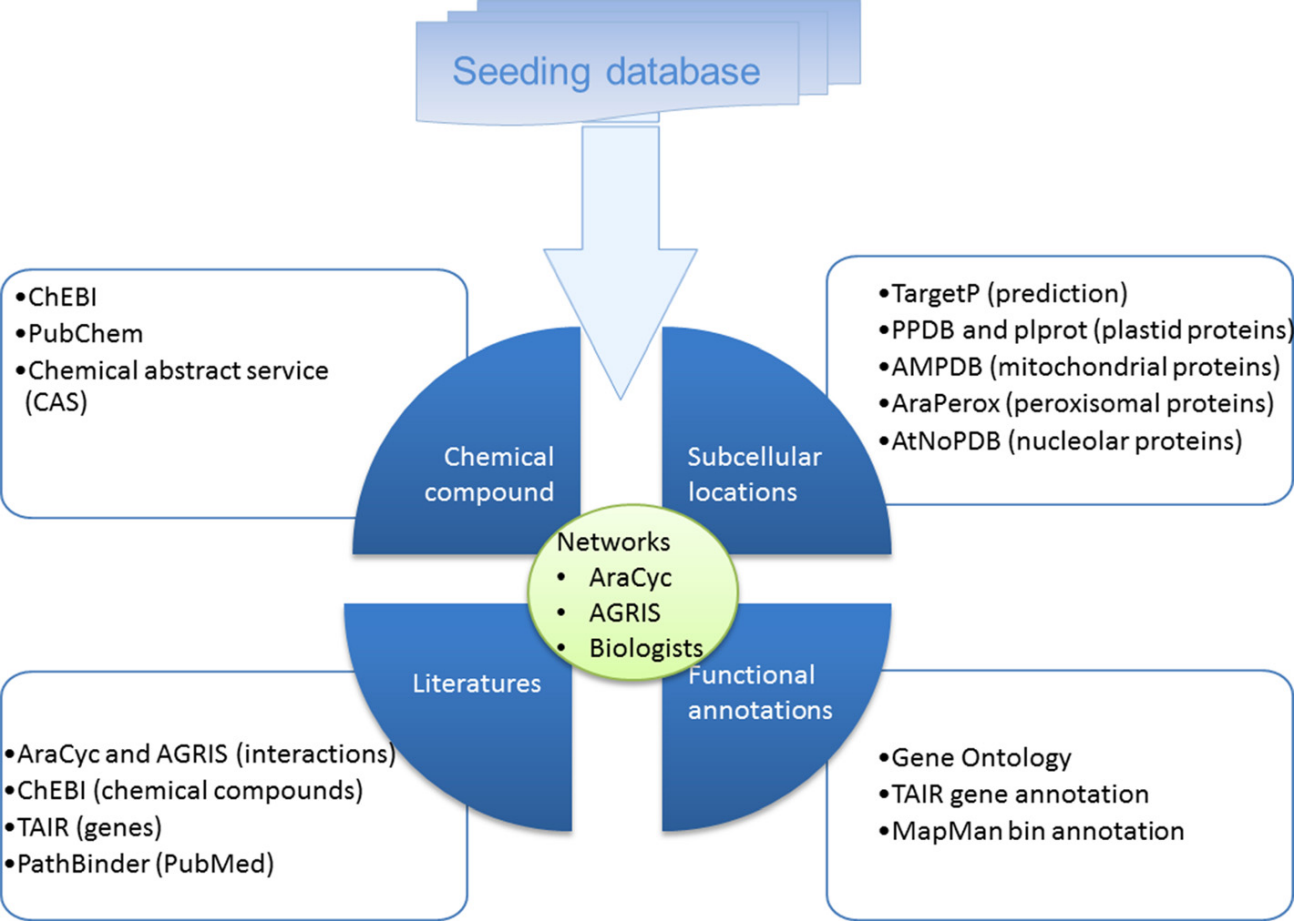
**The order of the data integration for MetNetDB, with respect to Arabidopsis: AraCyc is the backbone of the whole database, then AGRIS is used to extend the networks.** Other databases are integrated without any special order requirement. External data usually does not need any special transformation because the data can be easily added into to the database as a new field/label of a gene, a protein or a molecule. So the data integration from the external data source to MetNetDB is straightforward in most cases.

**Figure 2:**
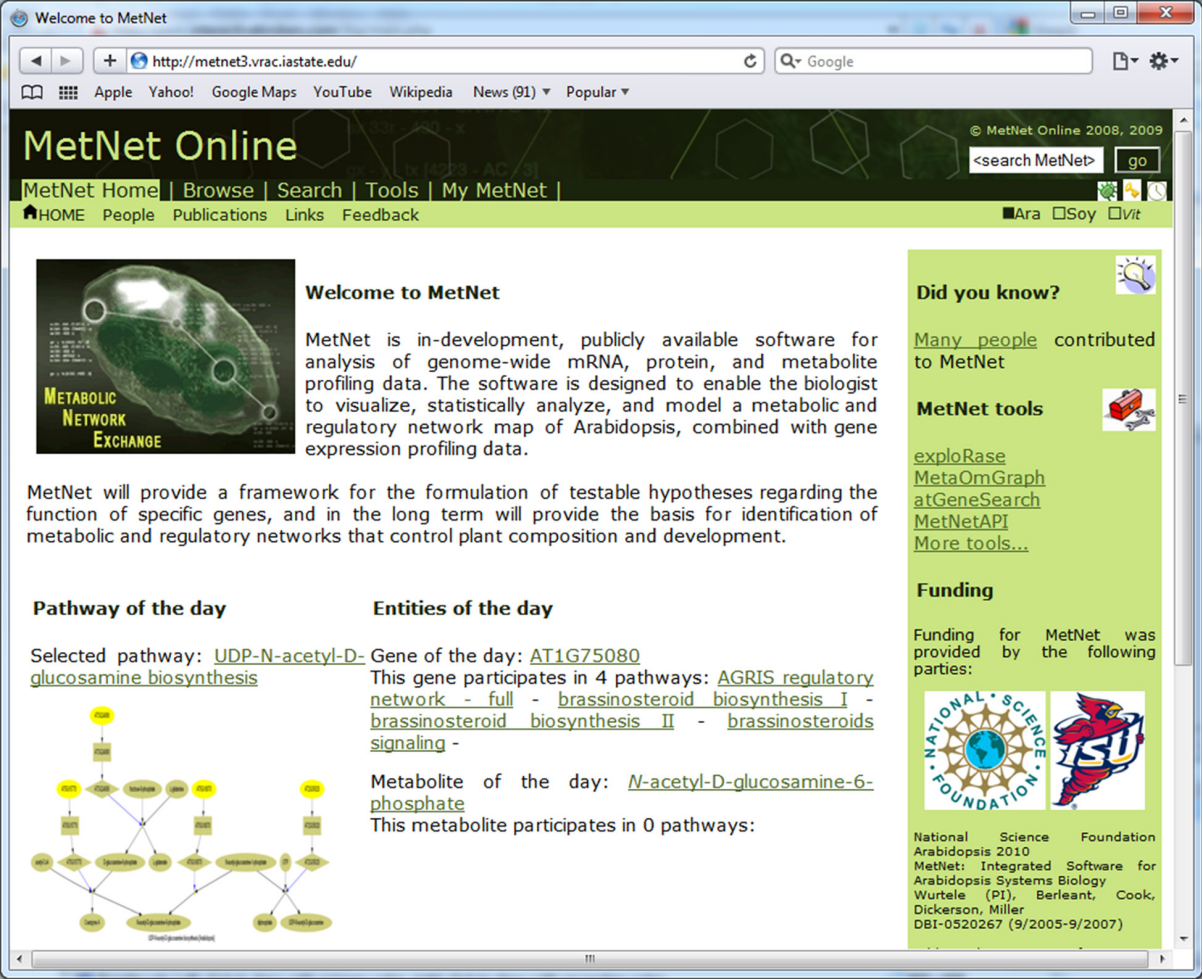
**The MetNet Online portal start page.** In order to rapidly familiarize novice users, a “pathway of the day” display and a “gene of day” display encourage self-guided exploration.

MetNet Online is a web application developed in PHP [29]. GraphViz ( http://www.graphviz.org) is used to generate graphical representations of pathways and networks (Figure 1). Biological entities and interactions are represented as nodes and the associations between them are represented as edges in the graph model. The database serves as the primary data repository for both our online portal and the MetNet suite of visualization and analysis tools [7]. Major features of the website are shown in Figure 2. A comparative list of features in relationship to select other online resources is available in Table 2.

MetNet Online is centred on several concepts that are inherent in the underlying database. Entities represent physical molecules (subtypes are DNA, RNA, protein, protein complex, and metabolite) and interactions can occur between any number of entities or between entities and other interactions (e.g., in the case of catalysis). A pathway is a collection of interactions that provides a convenient unit to represent prevailing cellular functions. Pathways in MetNet are predefined and cannot be changed by a user, but can be integrated with each other or with other interactions to form new units. A network in MetNet is a collection of interactions for which the granularity is determined by the user when (s) he creates it. A network can consist of any number of interactions or it can be a combination of some already defined pathways. It can also map to exactly one pathway or it can map to a pathway minus transcription/translation events. Networks are virtual and transient objects.

The database can be browsed based on different ontologies or navigation trees including pathway category (e.g., biosynthesis, respiration, and signal transduction), entity participation, cellular location (e.g., nucleus, plastid, and cytosol) and interaction type (e.g., diffusion, transport, and negative/positive regulation). After navigating through a tree and selecting a node of interest, a list of pathways is displayed (either in list-form or by thumbnail) and the pathway information screen can then be chosen or the pathway can be visualized directly. Visualization of a particular pathway may be different from what one is used to seeing at other locations or textbooks, as we rely on GraphViz’s algorithms to render the underlying graphs: no manual intervention (e.g. to make cycles more obvious) is currently implemented in our data flow.

Information about a pathway consists of general comments and literature references, location information, interactions contained within the pathway and participating entities. Sources and sinks for the pathway are displayed in a separate tab as part of the participating entities. This is critical information for simulations in which the pathway is treated as a black box (e.g., for the glycolysis-pathway, glucose would be a source and pyruvate would be a sink; ATP and ADP would serve as both source and sink). At the top-right of the pathway information screen, a toolbar is shown with export functions to various programs and a link to visualize the pathway (discussed separately). Entities can be browsed (alphabetically) independently of pathways and the entity information screen contains location information, possible synonyms, pathway participation and categorized interactions. Additional tabs are available on the pathway information screen.

The literature tab interfaces with PubMed to retrieve a current literature feed, in which the name of the pathway selected by the user is used as a search-term. Cellular context (i.e. the location within the cell where an entity is present when participating in an interaction) is represented separately, so that one can get an idea of the various roles a protein or metabolite might play. Another tab shows connected pathways that share one or more entities.

MetNet Online visualizes pathways with their known or predicted sub cellular locations. This information is not available anywhere else (out of 5527 proteins in AraCyc 8.0 e.g., only 286 have a location annotation). Sub cellular location information can help scientists develop hypotheses on gene function. Entities are color-coded according to assigned location and shape-coded according to entity type. Interactions are color-coded according to type for easy identification and visualization.

MetNet Online’s search function is integrated, rather than providing different search functions for entities, interactions and pathways. Thus, search operations for “regulation”, “biosynthesis”, “AT4G40090”, “AGP3” or “malate” use the same interface. Search results are grouped by entity types, interactions and pathways. When searching for “glucose”, for example, not only the “glucose” metabolite is presented but also the “glucose-UDP biosynthesis” pathway, among others. Synonyms are taken into account. Searches for “H2O” and “water” or “O2” and “oxygen” both lead to the same entity. Typos and misspellings result in suggestions that often point a visitor in the right direction and when no results are found for a search, potential alternatives are suggested. An example of this would be when “giberelin” is entered, the alternative “gibberellin” is proposed.

When visualizing a pathway, a GraphViz ( http://www.graphviz.org) .dot file is generated and transformed into its visual representation (dot layout). In the upper left corner of the screen, a thumbnail of the complete pathway is shown to allow easy navigation through complex maps. An indexed list of all participating entities is displayed underneath the thumbnail.

### Custom network design and personalization features

**Figure 3:**
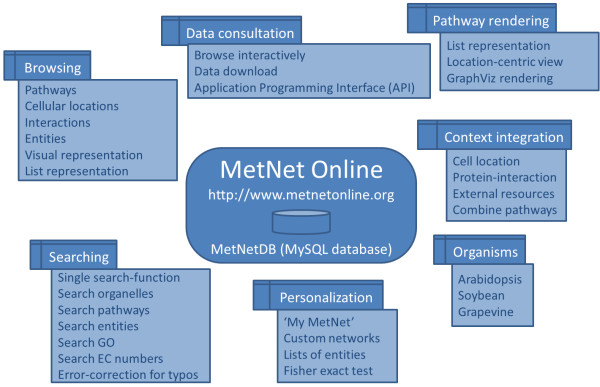
**Different browsing options.** Pathways can be browsed by the subcellular location where (part of) the pathway occurs. Hovering over a pathway in the right-side panel bring up a thumbnail of the pathway. The pathway can then be browsed in textual mode (enumerated list of interactions and entities that make up the pathway) and visual mode.

Most pathway databases are static; In contrast, MetNet Online represents all lists of interactions and pathways with checkboxes in front of them. A user can then choose either to visualize a pathway by itself or to select a set of pathways or interactions. Selection of any set pathways and/or interactions generates a new network by representing an integrated view of all selections (Figure 3). Due to computational and server restraints, we currently permit the user-generated network to consist of only up to 150 nodes. The result is visualized using the methods discussed earlier or it can be exported as a whole to other tools.

Any visitor can become a registered user of MetNet Online. This opens up access to the “My MetNet” function, which is implemented in a format that is similar to other personalization portals such as “My NCBI” or “My Yahoo”. When logged in, users gain access to additional functionality. Bookmarks can be used to efficiently retrieve objects of interest at any time in the future without having to navigate classification trees or execute a search. Entities, interactions and pathways can all be bookmarked, and bookmarked objects can have a commentary attached to them.

The “List of Entities” is a second function in “My MetNet”. Users can create and save multiple lists simultaneously. Lists can be created in three ways: a user can manually specify its members, convert a set of bookmarked entities into a list, or upload a text file. An entity list can include experimental data, such as over-represented or under-represented genes from transcriptomics analysis or metabolites from a GC/MS experiment. A list of entities, such as genes, or metabolites, can be forwarded to Reactome’s Skypainter function, to view an overlay of ‘omics data on the biological network [30,31]. Bird’s Eye View (BEV), part of the MetNet suite of applications and available through http://www.metnetdb.org, is another tool that can be used for this [7]. Privacy issues are taken into consideration. Data related to these lists is stored in the MetNetDB database, but is accessible only to the user that created it.

As a list gets longer, it is likely that additional pathways will be linked to the entities in that list. In order to put results in perspective and to distinguish relevant from less relevant pathways, a separate interface contains the results of Fisher’s exact test and ranks matching pathways by p-value (lesser values indicate higher relevance). Fisher’s exact test is available for both visitors and registered users. Registered userscan automatically apply the Fisher’s exact test to their stored customized lists of entities and interactions. Visitors have to specify their entities of interest manually in a text field. While a user can extract and visualize data from the network in a wide variety of ways, no user can change anything in the original network without going through a MetNet curator.

By providing the option to export pathways and subnetworks to other file formats, MetNet Online leverages existing software that incorporates a wide range of supplementary layout algorithms (CellDesigner [27], Cytoscape [32]) in a more suitable environment than the web browser. MetNet Online provides considerable connectivity for downstream data processing and it supports several export options including comma-separated values (CSV), SBML [33] and XGMML. SBML is sufficient to support most of the major features contained in the database and BioPax can be used to encode < annotation > −elements in the output [34,35]. As it becomes available, we plan to incorporate kinetic data in the SBML files . XGMML is useful because it allows MetNet Online data to be transferred to Cytoscape [32].

### Use cases

**Figure 4:**
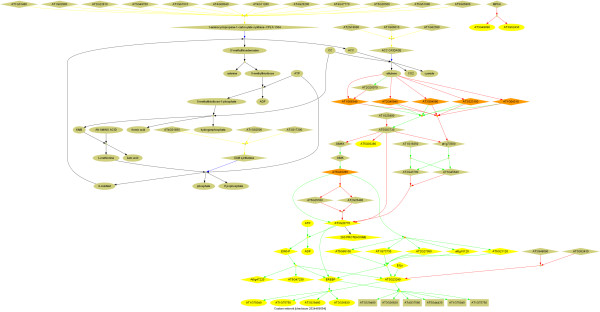
**A custom ethylene-related network.** The network was generated dynamically by selecting all pathways in which ethylene (ethane) was found.

A horticulturist who studies senescence is interested in ethylene metabolism and signalling [36]. She wants to look at what is known about the process in the model plant, Arabidopsis and visits the website and searches for the commonly used synonym,“ethene”. MetNet Online recognizes the synonym and includes “ethylene” in the list of search results. The user clicks on the link for the metabolite and sees three Arabidopsis pathways that involve ethylene. She selects all three and creates an integrated view. Although this is helpful in running additional analyses, she does not need the transcription and translation events. She logs into her “My MetNet” account and for easy access, she adds ethylene to her bookmarked entities. Using this shortcut, she visits the three ethylene-related pathways and examines the interactions contained in each pathway. She then bookmarks the interactions that are of particular interest to her. After doing so, she goes back to her bookmark overview page and sees a list of bookmarked interactions, all extracted from the various ethylene-related pathways. She asks for a new integrated view of only these bookmarked interactions and she uses the scaling function in the visualization module to observe the entire network. When she is satisfied, she clicks on the XGMML icon to export her custom network and transfers the data to Cytoscape [32], where she may examine additional properties of the network. The result is shown in Figure 4, and the entire scenario is described in more detail in an online video tutorial at http://www.metnetonline.org/tutorial.

In a second use case, a cell biologist has run a set of microarrays (or RNAseq) on developing soybean embryos. He identifies a list of differentially (under- and over-) expressed genes. He saves the probe-names (or gene names) as a separate text file (soybean_de.csv) and visits the MetNet Online website. He logs into his My MetNet account and creates a personalized list by uploading the text file. Because the probe-names are recognized by MetNet, he looks for pathways that are over-represented among the differentially expressed genes. Because numerous genes (thousands) are differentially expressed during embryo development, many pathways show up in an initial quantitative screen. As such, the user decides to use the Fisher exact test module to rank the pathway over-representation by p-value. This presents him with useful information; the list of pathways is still large but they are now ranked by p-value for relevance. He examines the pathways with the lowest p-values and is thus able to identify other potential gene targets for future experiments and verification. The two use case scenarios for MetNet are described in detail in an online video tutorial at http://www.metnetonline.org/tutorial.

## Conclusions

Molecular biologists, physiologists and biotechnologists aim to understand the function of particular genes, polypeptides or metabolites, and to develop testable hypotheses as to how these biological entities function to influence the overall biological network. Easy and convenient access to integrated information from a variety of biological data repositories can greatly facilitate these goals.

We have built a new portal, MetNet Online, which provides a gateway to integrative systems biology applications. The site generates simple pathways or complex representations of customized interaction sets or combined pathways.

In addition to existing data for Arabidopsis, soybean and grapevine biological networks, MetNet Online incorporates manually curated interactions, in particular signal transduction interactions, and also introduces a sub cellular location data layer. Our site supplements other previously created tools, and interfaces with many of them. Users can integrate pathways and interactions to build custom network and track objects (entities, interactions and/or pathways) that are of particular interest to them.

## Availability and requirements

The MetNet Online site can be reached at http://www.metnetonline.org. Extensive video tutorials on how to use the site are available through http://www.metnetonline.org/tutorial/.

## Competing interests

The author(s) declare that they have no competing interests.

## Authors’ contributions

YS conceptualized and implemented the web portal, and prepared this manuscript. YW was responsible for back-end data integration, data update, and data services. JL established the initial schema for the database and developed curation tools. ESW is principal investigator of this project and supervised this manuscript. All authors read and approved the final manuscript.

## Supplementary Material

Additional file 1 **Table S1.** Comparing MetNet Online with other resources. Click here for file
